# The role of miRNAs in viral myocarditis, and its possible implication in induction of mRNA-based COVID-19 vaccines-induced myocarditis

**DOI:** 10.1186/s42269-022-00955-1

**Published:** 2022-11-17

**Authors:** Antoine AbdelMassih, Hala Agha, Sonia El-Saiedi, Amal El-Sisi, Meryam El Shershaby, Hanya Gaber, Habiba-Allah Ismail, Nadine El-Husseiny, Abeer Reda Amin, Aly ElBoraie, Aya Ayad, Esraa Menshawey, Fady Sefein, Ibrahim Ihab Osman, Mai Moursi, Maram Hanafy, Mariam Sherif Abdelaziz, Mariem Badr Arsanyous, Mariam Khaled-Ibn-El-Walid, Marwa Gamal Tawfik, Menna Habib, Mina Ehab Mansour, Mirette Ashraf, Mohamed Ayman Khattab, Nada Alshehry, Nada Hafez, Naheel Essam ElDeeb, Nirvana Ashraf, Noha Khalil, Noheir Ismail AbdElSalam, Noura Shebl, Nouran Gamal Ali Hafez, Nourhan Hatem Youssef, Odette Bahnan, Passant Ismail, Peter Kelada, Rahma Menshawey, Rana Saeed, Reem Jalal Husseiny, Reem Yasser, Safa Sharaf, Veronia Adel, Youstina Naeem, Youstina Nagy Farid Nicola, Aya Kamel, Rafeef Hozaien, Raghda Fouda

**Affiliations:** 1 Pediatric Cardiology Unit, Pediatrics’ Department, Faculty of Medicine, Cairo University, P.O. Box 12411, Cairo, Egypt; 2 Pediatric Cardio-Oncology Clinic, Children Cancer Hospital of Egypt, Cairo, Egypt; 3 Student and Internship Research Program (Research Accessibility Team), Faculty of Medicine, Cairo University, Cairo, Egypt; 4 Faculty of Dentistry, Cairo University, Cairo, Egypt; 5 Pixagon Graphic Design Agency, Cairo, Egypt; 6 Clinical and Chemical Pathology Department, Faculty of Medicine, Cairo University, Cairo, Egypt

**Keywords:** Micro-RNA, mRNA COVID-19 vaccines, Myocarditis, Sarcopenia

## Abstract

**Background:**

Several reports of unheeded complications secondary to the current mass international rollout of SARS-COV-2 vaccines, one of which is myocarditis occurring with the FDA fully approved vaccine, Pfizer, and others.

**Main body of the abstract:**

Certain miRNAs (non-coding RNA sequences) are involved in the pathogenesis in viral myocarditis, and those miRNAs are interestingly upregulated in severe COVID-19. We hypothesize that the use of mRNA-based vaccines may be triggering the release of host miRNAs or that trigger the occurrence of myocarditis. This is based on the finding of altered host miRNA expression promoting virus-induced myocarditis.

**Short conclusion:**

In conclusion, miRNAs are likely implicated in myocarditis associated with mRNA vaccines. Our hypothesis suggests the use of miRNA as a biomarker for the diagnosis of mRNA vaccine-induced myocarditis. Additionally, the interplay between viral miRNA and the host immune system could alter inflammatory profiles, hence suggesting the use of therapeutic inhibition to prevent such complications.

## Background

Many lives were lost since the emergence of COVID-19 (coronavirus disease 2019) pandemic in China 2019. The resultant global health crisis has compelled health authorities all over the world to allow emergency authorization to many vaccines before full FDA (Food and drug agency) approval. However, a major setback of such emergency authorization was the rise of unexpected complications.

One of the most serious complications of the COVID-19 vaccines is post-vaccination myocarditis associated with the mRNA vaccines (Krause and Gruber 2020).

According to the US centers for disease control and prevention, the myocarditis/pericarditis rate is 12.6 per million doses of second dose mRNA vaccine among individuals 12–39 years of age (Bozkurt et al. 2021).

There were no reported cases of myocarditis with non-mRNA (messenger ribonucleic acid) vaccines such as Janssen. Patients who developed myocarditis presented with chest pain and elevated troponin levels after receiving the second dose of the mRNA vaccine. Most of their symptoms resolved without treatment (Bozkurt et al. 2021).

The mechanism of this relationship is still not fully understood yet. However, the trending hypothesis is that mRNA vaccines trigger an antibody response similar to Multi-System Inflammatory Syndrome in children. Another hypothesis is the molecular mimicry between SARS-CoV-2 (severe acute respiratory syndrome causing coronavirus-2) proteins and self-antigens, triggering an abnormal immune response reaction (Pillay et al. 2022).

In the last decade, there has been increasing evidence supporting the fact that dysregulated miRNA (micro-Ribonucleic Acid) expression mediates the pathogenesis of viral myocarditis. miRNAs are short non-coding RNAs that regulate gene expression during cellular processes, and they are known to mediate inflammation, myocardial remodeling, and reverse remodeling. They are either host-generated after exposure of tissues to viral DNA/RNA or directly derived from the manipulation of the host cellular machinery to viral DNA and RNA.

These two sources of miRNAs might be hypothetically induced by mRNA vaccines and might mean that recognizing and screening certain miRNA levels following administration of mRNA vaccines might help in predicting the occurrence of mRNA vaccine-induced myocarditis. (O’Brien et al. 2018).

It was also recognized that some of those miRNAs were negatively regulated with aging, due to loss of muscle mass. This fact might explain the age likelihood of mRNA vaccine-induced myocarditis which tends to affect adolescents rather than individuals older than 40 years of age (Ong et al. 2019).

Surprisingly to date, no study has explored the serum levels of target miRNAs known to induce viral myocarditis in patients developing mRNA vaccine-induced myocarditis. In this article, we are shedding light on the main miRNAs involved in the induction of viral myocarditis, the main mechanisms involved in their synthesis, as well as the main therapeutics blocking their deleterious effects. This might draw the attention of the scientific community toward testing those target miRNAs in individuals developing mRNA COVID vaccine-induced myocarditis, hence tailoring some predictors and therapeutics to this serious complication.

## Main body

### Sources of miRNAs

As mentioned earlier, there are two main sources generating miRNAs. Both sources are a result of the interplay between host and viral DNA or mRNA. Either viral DNA or RNA upregulate or downregulate some key host-generated miRNAs or the host enzymes derive miRNAs from the viral DNA or RNA.

-Several host miRNAs have a direct relationship with myocarditis and are either upregulated or downregulated by viruses causing myocarditis. 94 types of miRNAs were studied using gene microarray analysis in the setting of viral myocarditis; myocarditis downregulated 27 types and upregulated 67. One of the miRNAs, namely miRNA214, promotes cardiac inflammation by increasing the expression of TNF alpha and IL-6 (tumor necrosis factor and interleukin-6). Its level is elevated in plasma during myocarditis and could be used as a noninvasive biomarker for diagnosing myocarditis (Wang and Han 2020).

Studies suggest that miRNA regulation could also have a therapeutic role in myocarditis. For instance, in coxsackievirus B3 (CVB3)-induced viral myocarditis, macrophage infiltration and miRNA-155, which regulates the differentiation of macrophages, were found to be hallmark features of viral myocarditis. miRNA-155 silencing might affect macrophage polarization and shifting of the inflammatory mediator's balance, resulting in an increase in alternatively activated macrophages (M2) and a decrease in classically activated macrophages (M1) in the heart. Subsequently, the risks associated with viral myocarditis decrease. Hence, antagonizing miR-155 is a potential therapeutic target against viral myocarditis (Mansour et al. 2021; Minocha et al. 2021).

Another study by Corsten et al. revealed that anti-miRNA-155 therapy in viral myocarditis decreased monocyte-macrophages levels, suppressed T-lymphocyte activation, and inhibited the release of pro-and anti-inflammatory cytokines (including TNF- α, Il-6, IL-10, and IFN- γ[Interferon]) during the inflammatory phase. Furthermore, suppression of miRNA-155 reduced weight loss caused by systemic illness and reduced the number of circulating leukocytes induced by CVB3 infection (Corsten et al. 2012).

The role of miRNA-21 in viral myocarditis is controversial, and Xu and colleagues proved that upregulation of miRNA-21 in myocarditis leads to overexpression of mitogen-activated protein kinase, thereby increasing myocardial fibrosis and mediation of the transition from myocarditis to dilated cardiomyopathy. CVB3-upregulated miRNA-21 can cause cardiomyocyte damage by disruption of their cell-to-cell interactions. In the setting of CVB3 infection, miRNA-21 expression can cause VMC, and its suppression can reduce host injury (Xu et al. 2014).

Ye et al. confirmed the deleterious effect of miRNA-21 by showing a disruption of cardiac intercalated disks through its upregulation, resulting in myocardial damage and worsening the outcome of myocarditis (Xu et al. 2014; Ye et al. 2014).

On the contrary, He et al. stated that CVB3 reduced the expression of miRNA-21, which directly inhibits programmed cell death 4 and worsens myocarditis as a result of prolonging cell survival and viral replication (He et al. 2013). More studies are needed to elucidate protective and deleterious levels of tissue and serum levels of miRNA-21.

Another strategy involved in the pathogenesis of viral myocarditis is enhancing initial cell survival, hence promoting viral replication. To sum up, CVB3 tends to induce miRNAs which balances cardiac cell survival versus apoptosis and therefore creates an optimal environment for viral replication. Interestingly, miRNA-590-5p released extracellularly by CVB3 inhibits pro-apoptotic factors, resulting in prolonged viral replication. miRNA-98, which regulates cell apoptosis through the FAS/FASL gene pair, was found to be downregulated in myocarditis patients. (Wu et al. 2015). It can also suppress IL-10 (a mediator derived from B-cells) and limit the severity of myocarditis; hence, its downregulation increases the yield of pro-inflammatory cytokines (Corsten et al. 2012).

miRNA-29 also has a deleterious effect in VMC. It was found to prolong cell survival and enhance viral replication. It also promotes collagen deposition and hypertrophy of cardiomyocytes (Zhu et al. 2021).

Speaking of miRNA125b, which stimulates collagen synthesis, it also acts as a repressor of cardiac antifibrotic mechanisms, and thus its inhibition could be a promising therapeutic target in dilated cardiomyopathy (Krützfeldt et al. 2005).

In a study suggesting a potential treatment for myocarditis, RT-qPCR showed reduced miRNA-141-3p expression in mice with experimental autoimmune myocarditis (EAM). miRNA-141-3p is known to decrease myocardial inflammation by suppressing STAT4 (Wang et al. 2018).

Scientists injected mice with miRNA-141-3p agomir to test its effect on the pathology of EAM. The results showed reduced LVEDd and LVEDs (left ventricular end-diastolic and end end-systolic dimensions) on echocardiography in the non-injected mice, and increased LVEF and LVFS (Fractional shortening) in miR-141-3p agomir-injected mice. MiRNA-141-3p overexpression (Buscaglia and Li 2011).

As mentioned earlier, miRNAs profiles have not been studied yet in recipients of mRNA COVID-19 vaccines developing myocarditis. However, interestingly many of the key miRNAs involved in induction of viral myocarditis are also upregulated in severe COVID-19. This shared profile might signify that SARS-CoV-2 antigens or RNA might upregulate the same miRNAs implicated in induction of myocardial inflammation.

Table 1 summarizes the effects of micro-RNAs released during myocarditis and severe COVID-19.

**Table 1:** Shared profile of miRNAs in VMC and severe COVID-19

miRNAs	Role in viral myocarditis	Overall effect in viral myocarditis	References	State in severe COVID-19	References
miRNA-155	Regulates macrophages differentiation by increasing type 1 macrophages and decreasing type 2, thus increasing pro-inflammatory cytokines	Deleterious	Minocha et al. (2021)	Increased in severed COVID-19, and can be used as a potential biomarker of mortality	Haroun et al. (2022)
miRNA-21	Protective effect: Pro-apoptotic thus interrupting cell survival and viral replication Deleterious effect: Disrupts cardiac intercalated disks	Controversial	Buscaglia and Li (2011), He et al. (2013), Xu et al. (2014), Ye et al. (2014)	Increased levels linked to ICU admission	Calderon-Dominguez et al. (2022)
miRNA-98	Pro-apoptotic thus interrupting cell survival and viral replication Regulates cell apoptosis, Suppresses pro-inflammatory IL-10	Protective	Corsten et al. (2012)	Suppresses TMPRSS2 and inhibits cellular entry of SARS-CoV-2	Matarese et al. (2020)
miRNA590-5p	Inhibits pro-apoptotic factors, prolong cell survival and increases viral replication	Deleterious	Wu et al. (2015)	Suppresses Type I interferon and this leads to decreased innate immune response to COVID-19	Farr et al. (2021)
miRNA-29b	Inhibits pro-apoptotic factors, prolong cell survival, and increases viral replication Induces myocardial fibrosis	Deleterious	Zhu et al. (2021)		
miRNA125b	Induces myocardial fibrosis	Deleterious	Krützfeldt et al. (2005)		
miRNA141-3p	Decreases myocardial inflammation by suppressing STAT4	Protective			

*COVID-19* Coronavirus disease 2019, *miRNA* micro-ribonucleic acid, *ICU* Intensive Care Unit, *IL* Interleukin, *SARS-CoV-2* severe acute respiratory syndrome coronavirus disease 2, *STAT4* Signal transducer and activator of transcription 4, *TMPRSS2* transmembrane serine protease 2, *VMC* viral myocarditis

-In the last decade, multiple DNA and RNA viruses were shown to produce miRNAs known as viral miRNAs (v-miRNAs) to evade the host immune response.

*More than 250 v-miRNAs* exist. The majority of which are present in the DNA viruses of the herpes virus family. The detection of v-miRNAs in RNA viruses is controversial. A few reports suggested that non-canonical miRNA-like small RNAs are produced during RNA virus infections. However, these small RNAs lack the canonical stem-loop structure of miRNAs, and thus their biogenesis and function are not well understood (Pan et al. 2019).

The following reasons might explain the lack of v-miRNAs produced by RNA viruses during infection*:* (a) the RNA viruses consist of positive or negative sense or double-stranded RNA (dsRNA) and replicate in the host cell cytoplasm, which is inaccessible to the miRNA biogenesis machinery in the nuclei. (b) Excision of pre-miRNA from the primary transcript might destroy RNA-based viral genomes. c-the generated v-miRNA may target the viral genome itself*, *cleaving the viral genome. To date, viruses involved in myocarditis were not found to release such types of v-miRNAs. However, below are the main cellular effects of the currently discovered v-miRNAs (Li and Zou 2019).

*Improving cell survival: A classic example is EBV (Epstein Barr Virus) miRNA-BART5 (Bamhi fragment A rightward transcript), which controls proliferation and establishes latent infection by targeting PUMA. PUMA (The p53 upregulated modulator of apoptosis) is known to modulate apoptosis by p53. So, by suppressing PUMA, EBV miRNAs alter the susceptibility to apoptotic agents and improve host cell survival (Chen et al. 2017).

*Altering cytokine expression: KSHV (Kaposi sarcoma/herpes virus) v-miRNAs reduce the expression of C/EBPβ p20 (Enhancer binding protein), a known negative regulator of IL-6 and IL-10 cytokines. This results in regulation of cytokine signaling in infected cells and overexpression of pro-inflammatory IL-6 leading to tissue damage (Yang et al. 2018).

*Altering antiviral immune responses: HCMV (Human cytomegalovirus) miRNAs target host genes involved in the antiviral immune response. miRNA-UL112 blocks the natural killer (NK) cell-mediated recognition of virus-infected cells, by inhibiting MICB expression (MHC class I chain-related protein B), which is a stress-induced ligand essential for NK-cell activity (Nanbakhsh and Malarkannan 2020).

Table 2 summarizes miRNAs released by different DNA and RNA viruses.

**Table 2:** DNA and RNA viruses producing miRNAs and the numbers of miRNAs discovered for each

Virus family	Type	Number of encoded pre-miRNAs and miRNAs	References
γ-Herpesvirus	DNA	40 pre-miRNAs and 4 mature miRNAs	Pan et al. (2019), Najarro et al. (2015), Chen et al. (2017)
β-Herpesvirus	DNA	26 miRNAs related mainly to HCMV	
α-Herpesvirus	DNA	24 functional miRNAs	
Papillomavirus	DNA	Four (two by HPV16, one by HPV38, and one by HPV68)	
Hepadnavirus	DNA	only one	
Adenovirus	DNA	encodes two miRNAs	
Polyomavirus	DNA	one pre-miRNA at the 3′ end that encodes two mature miRNAs	
Influenza Virus	RNA	encodes small viral leader miRNAs	
Ebola Virus	RNA	seven mature miRNAs from four pre-miRNAs	
HIV-1	RNA	five putative pre-miRNAs	

*DNA* Deoxy-Ribonucleic acid, *HCMV* Human Cytomegalovirus, *HIV* Human immunodeficiency virus, *HPV* Human Papilloma Virus, *miRNA* Micro Ribonucleic acid

### How can the miRNAs hypothesis explain the age likelihood of mRNA COVID-19 vaccines-induced myocarditis?

The inflammatory and molecular mimicry hypotheses explaining the mechanism of myocarditis secondary to COVID-19 mRNA vaccines failed to justify why old children and young adolescents are more prone to the observed complication. Aging should increase the baseline cytokine levels and the serum and tissue levels of pro-inflammatory cytokines, a process known as inflame-aging, which should theoretically imply an increase in myocarditis from mRNA vaccines in older age individuals. However, our miRNAs hypothesis interestingly matches the age likelihood of this complication (Franceschi et al. 2018).

Key miRNAs involved in the induction of myocarditis are expressed in skeletal muscles such as miRNA-155. Sarcopenia or loss of muscle mass is mostly pronounced after 50 years of age, occurring steadily at a rate of 1–2% annually. This loss of muscle mass correlates with levels of decreased micro-RNAs, particularly miRNA-155. This also might explain why males, with higher muscle mass compared to females, are more likely to develop myocarditis following mRNA vaccines (Ong et al. 2019).

### Implications:

#### Diagnostic: predicting mRNA-vaccines-related myocarditis

*As mentioned earlier, viral RNA can either alter the expression of host miRNA or use cellular machinery to form viral miRNAs. Farr and colleagues have used next-generation sequencing to determine miRNAs implicated in severe COVID-19 (Farr et al. 2021). Many miRNAs implicated in severe COVID-19 are potential targets for viral myocarditis. Screening for such miRNAs serum levels can determine the likelihood of recipients of mRNA vaccines to develop myocarditis. The cost-effectiveness is not certain, given the relative rarity of the complication (Mishra et al. 2020).

*The previous data suggest that increased muscle mass might increase myocarditis in individuals at higher risk of myocarditis from mRNA vaccines by the sarcopenia index. The higher the index, the lower the risk of myocarditis from mRNA vaccines. Screening of the non-costly sarcopenia index in recipients of mRNA might help to determine who is at higher risk to develop myocarditis (Ong et al. 2019).

#### Therapeutic targets

*The miRNA-132-3p is a regulatory (non-coding) RNA that is upregulated in cardiac tissue in response to cardiomyocyte inflammation. It affects signaling pathways involved in cardiomyocyte growth, autophagy, calcium handling, and contractility. Thus, miRNA-132 appears as a potentially promising molecular pathophysiological target in myocarditis and heart failure (HF) treatment. CDR132L is a miRNA-132 inhibitor. It is a synthetic nucleic acid antisense oligonucleotide (ASO) inhibitor with a phosphorylated backbone.

A prospective, randomized, double-blind, placebo-controlled, dose-ranging study of intravenous antagonist of miRNA-132-3p (synthetic antisense oligonucleotide as a pharmacological inhibitor of miRNA-132) was performed by Täubel and colleagues on 28 HF patients, with left ventricular ejection fraction between ≥ 30% and < 50%, or amino-terminal fragment of pro-brain natriuretic peptide (NT-proBNP) > 125 ng/L at screening, and they were randomized to receive CDR132L (0.32, 1, 3, and 10 mg/kg body weight) or placebo (0.9% saline). Dose-dependent CDR132L treatment reduced miRNA-132 in plasma. Patients given CDR132L ≥ 1 mg/kg displayed a median 23.3% NT-proBNP reduction, versus a 0.9% median increase in the control group. CDR132L treatment induced significant QRS narrowing and encouraged positive trends for relevant cardiac fibrosis biomarkers. CDR132L was safe and well tolerated, without dose-limiting toxicity. The suggested dose level is 1 mg/kg of CDR132L. Both, direct inhibition and indirect inhibition of miRNAs were proven useful (Rohani et al. 2021).

*Pirfenidone is a small oral antifibrotic agent that inhibits the activation of cardiac fibroblasts and the production of peptides, such as transforming growth factor-β. It also works through antisense oligonucleotides directed against cardiotropic long non-coding RNA (miRNA, lncRNA). It silences miRNA-21 that promotes cardiac fibrosis, diminishing fibroblast proliferation, and collagen type 1 production and crosslinking that cause heart fibrosis.

In a randomized clinical trial conducted by Gavin A Lewis et al., to evaluate the efficacy of pirfenidone in the treatment of heart failure patients with preserved ejection fraction, pirfenidone and placebo were compared in the treatment of HFpEF (Heart Failure with preserved Ejection Fraction) in 47 participants of mean age 78 and mean myocardial extracellular volume 30%, shown by cardiac imaging 30%.

The results showed a reduction in myocardial fibrosis in 52 weeks. 100 capsules of pirfenidone were taken and resulted in a mean reduction of 0.06%. There was an improvement in 8 out of 10 KCCQ (Cardiomyopathy Questionnaire (Kansas City scores)), including clinical improvements and log NT-proBNP reduction.

The reduction in log NT-proBNP with pirfenidone was due to left ventricular myocardial stiffness improvement secondary to myocardial fibrosis regression. The decrease in myocardial fibrosis with log NT-proBNP reduction provided further evidence of its effect in decreasing heart fibrosis and treating HFpEF through non-coding miRNA silencing. The drug is effective in the structure but not the hemodynamic changes occurring in Heart failure. So, RAAS (renin–angiotensin–aldosterone system) inhibitors should be studied for cardiac hemodynamic effects, synergistically with pirfenidone (Aimo et al. 2020).

*Another modulator of miRNA levels is using RNA helicase; Wang and colleagues studied the effect of RNA helicases on miRNA expression in breast tissue and found similar effects of RNA helicases to miRNA antagonists on tissue levels of deleterious miRNA-21 involved in the development of breast cancer (Wang et al. 2012).

**Fig. 1:**
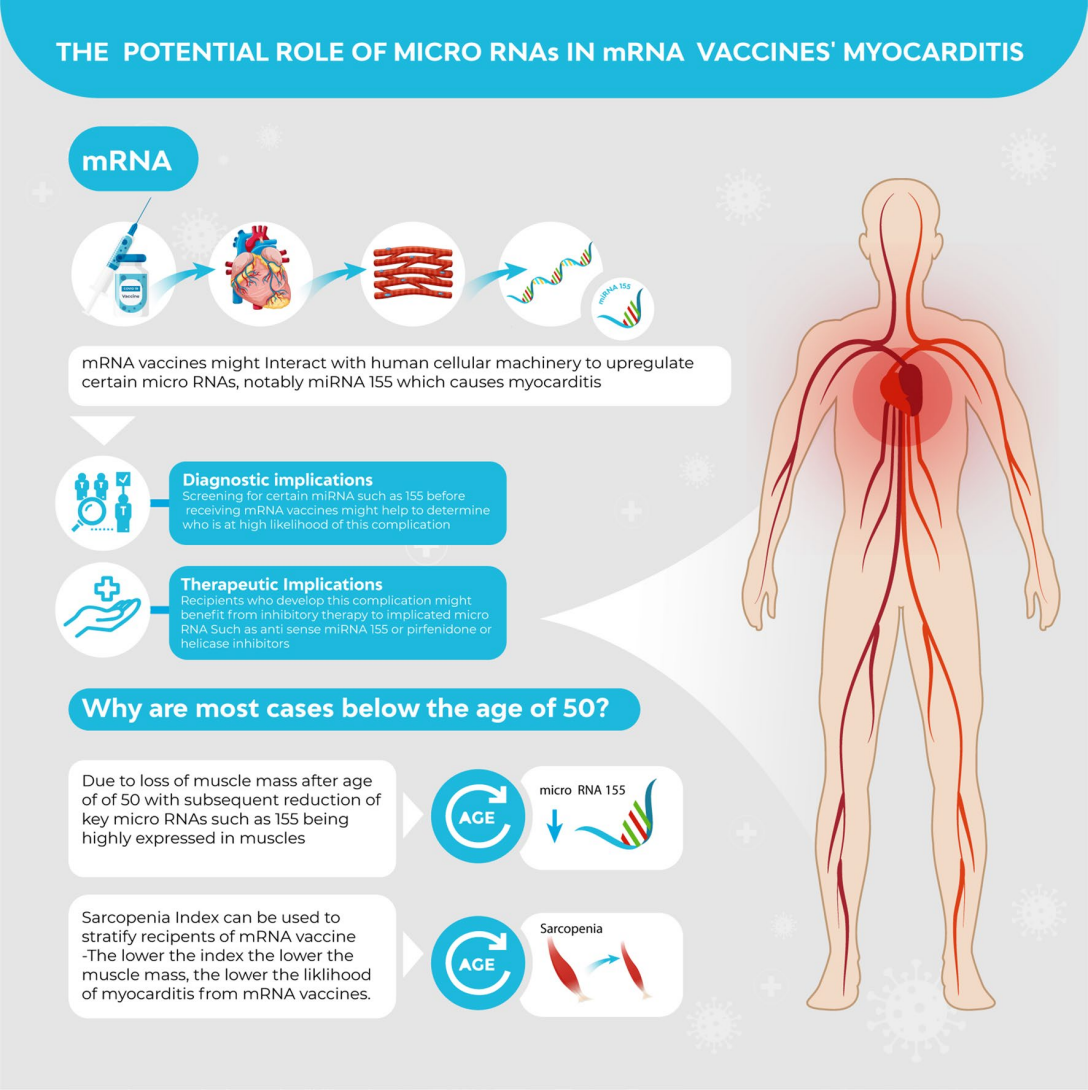
The potential role of micro-RNAs in mRNA-based vaccines. References: 1**–**56 as it is a summary of the article data

Figure 1 summarizes the implications discussed in this article.

## Conclusions

In conclusion, miRNA upregulation or downregulation by mRNA vaccines might explain the myocarditis observed from mRNA vaccines. This might explain why younger male individuals are at a higher risk of myocarditis as they have larger muscle mass, expressing higher circulating levels of myocarditis-producing miRNAs. Key miRNAs, such as miRNA-155, are highly expressed in skeletal muscles. Recognizing this fact allows risk stratification of this complication before receiving mRNA vaccines, via sarcopenia index calculation or testing for target miRNAs serum levels. miRNAs can also be therapeutic by developing antisense miRNAs blocking the action of the implicated miRNAs and thus preventing the myocarditis process.

It is also to be noted that there is a trend toward the future use of mRNA technology for developing vaccines against other infectious agents and cancer. The early identification of the mechanisms involved in the pathogenesis of complications induced by those vaccines can help in avoiding and treating similar complications when those vaccines are targeting other disorders such as HIV or malignancies (Pardi et al. 2018).

## Data Availability

Not applicable.

## Abbreviations

AMOs: Anti-miRNAs Oligonucleotides
Ang 2: Angiotensin 2
AR: Androgen Receptor
ASO: Antisense Oligonucleotide
Bcl-2: B-cell Lymphoma 2
Bcl-xl: B-cell Lymphoma extra large
CMV: Cytomegalovirus
COVID-19: Corona Virus Disease-2019
CVB3: Coxsackie Virus B3
DCM: Idiopathic Dilated Cardiomyopathy
E2CI: Enterovirus-2C inhibitor
EBV: Epstein Barr Virus
ECM: Extracellular Matrix
FDA: Food and Drug Administration
HF: Heart Failure
HFpEF: Heart Failure preserved Ejection Fraction
HIT: Heparin-induced Thrombocytopenia
IFN: Interferon
IL: Interleukin
KSHV: Kaposi Sarcoma-like Herpes Virus
LNA: Locked Nucleic Acid
LVED: Left Ventricular End Diastolic
LVEF: Left Ventricular Ejection Fraction
LVFS: Left Ventricular Fractional Shortening
miRNA: Micro-RNA
mRNA: Messenger RNA
NK: Natural Killer
NT-proBNP: Amino Terminal Fragment of pro-Brain Natriuretic Peptide
PCR: Polymerase Chain Reaction
PDCD4: Programmed Cell Death 4
RAAS: Renin Angiotensin Aldosterone System
REST: Repressor Element 1 Silencing Transcription
SARS: Severe Acute Respiratory Syndrome
SOCS-3: Suppressor of Cytokine Signaling3
TNF: Tumor Necrotic Factor
TUG: Taurine Up-regulated Gene
VMC: Viral Myocarditis
